# Adverse impact of acute *Toxoplasma gondii* infection on human spermatozoa

**DOI:** 10.1111/febs.70097

**Published:** 2025-05-03

**Authors:** Lisbeth Rojas‐Barón, Leandro Tana‐Hernandez, Mireille H. Nguele Ampama, Raúl Sanchéz, Ulrich Gärtner, Florian M. E. Wagenlehner, Christian Preußer, Elke Pogge von Strandmann, Carlos Hermosilla, Anja Taubert, María E. Francia, Zahady D. Velasquez

**Affiliations:** ^1^ Institute of Parasitology Justus Liebig University Giessen Germany; ^2^ Laboratory of Apicomplexan Biology Institut Pasteur de Montevideo Uruguay; ^3^ Center of excellence in Translational Medicine—Scientific and Technological Bioresource Nucleus (CEMT—BIOREN), Faculty of Medicine Universidad de la Frontera Temuco Chile; ^4^ Department of Preclinical Sciences, Faculty of Medicine Universidad de la Frontera Temuco Chile; ^5^ Institute of Anatomy and Cell Biology Justus Liebig University Giessen Germany; ^6^ Clinic for Urology, Pediatric Urology and Andrology Justus Liebig University Giessen Germany; ^7^ EV – Core Facility, Institute for Tumor Immunology, Center for Tumor Biology and Immunology (ZTI) Philipps University Marburg Germany; ^8^ Department of Parasitology and Mycology, School of Medicine Universidad de la Republica Montevideo Uruguay

**Keywords:** acephalic spermatozoa, human spermatozoids, infertility, *Toxoplasma gondii*

## Abstract

*Toxoplasma gondii* is an obligate intracellular protozoan parasite that can infect virtually any nucleated cell within human and other endoderm animal tissue, including male reproductive organs. Herein, we investigate the capacity of *T. gondii* tachyzoites to infect and proliferate within the testes and epididymis and examine the resulting impact on human spermatozoa structure and functionality. We confirmed that *T. gondii* tachyzoites colonise and proliferate within the testes and epididymis, altering the tissue structural homeostasis, and causing immune cell infiltration and cellular damage. In addition to demonstrating that *T. gondii* remains infective within the testes and epididymis, *in vitro* experiments demonstrated a direct interaction between *T. gondii* tachyzoites and human spermatozoa. This resulted in a significant proportion of headless spermatozoa. Scanning and transmission electron microscopy revealed structural defects in spermatozoa, such as twisted tails and plasma membrane disruptions. Moreover, *T. gondii* tachyzoites triggered the loss of mitochondrial membrane potential (MMP) in spermatozoa without modulating reactive oxygen species (ROS) concentrations, and triggered cell death, pointing at mitochondrial dysfunction as a potential mechanism mediating spermatozoan damage. Our findings suggest that *T. gondii* infection can have profound implications for male fertility by directly damaging spermatozoa and altering testicular and epididymal structures. The study underscores the need for further research to elucidate the long‐term impact of *T. gondii* on male reproductive health, particularly in the context of iatrogenic infertility. Given the widespread seroprevalence of *T. gondii* in the human population, our research emphasises the importance of considering parasitic infections in diagnosing and managing male infertility in the field of andrology.

## Graphical abstract

*Toxoplasma gondii* tachyzoites injected intraperitoneally crossed the blood‐testes barrier, appearing in testes/epididymis by day 6 post‐infection. *In vitro* exposure caused increased sperm necrosis/apoptosis and structural damage to heads/tails. Immunofluorescence and SEM revealed tachyzoites attaching to sperm tails, inducing twisting/detachment, and creating head perforations. These findings suggest *T. gondii* infection may impair male fertility through direct sperm damage.

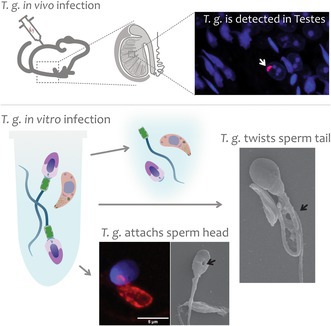

**Abbreviations**
ASS: acephalic spermatozoa
BBB: blood–brain barrier
BTB: blood‐testes barrier
HTCA: head‐tail coupling apparatus
MMP: mitochondrial membrane potential
MOI: multiplicity of infection
n.c.: negative control
p.c.: post‐confrontation
p.i.: post‐infection
PI: propidium iodide
ROS: reactive oxygen species
SEM: scanning electron microscopy
TEM: transmission electron microscopy

## Introduction

*Toxoplasma gondii* is an obligate intracellular apicomplexan parasite that poses serious health risks for both people and animals. Prenatal infections can result in abortion or have a significant impact on the offspring's welfare, particularly in humans and sheep [1]. Acute *T. gondii* infections in immunocompromised humans can be fatal, while chronic/latent infections have the potential to reactivate upon decay in immunity. This infection is notably prevalent in the human population. Global estimates suggest that 25–50% of the worldwide population is a chronic carrier of the parasite [2, 3]. Worldwide seroprevalence varies largely, with rates that range between 0.5% and 87% according to the region of the world analysed, whereby Africa, South America and Oceania display the highest average seroprevalence rates. Felines, including domestic cats, are the parasite's definitive hosts, which shed unsporulated oocysts to the environment. Following sporulation, oocysts are ingested by intermediate hosts, such as humans, where the parasite develops into fast‐replicating tachyzoites, causing the acute phase of toxoplasmosis. *T. gondii* can persist in several tissues such as the brain, muscles, eyes or lungs for the duration of the host's life by forming tissue cysts containing slow‐replicating bradyzoites [4]. Ingestion of tissue cysts through carnivorism allows horizontal transmission among intermediate hosts. Finally, *T. gondii* can be transmitted vertically, from mother to foetus.

Albeit rare, testicular toxoplasmosis has been documented in an immunocompetent 26‐year‐old male with chronic testicular granulomatous inflammation [5]. These findings are surprising since life, vital organs structures in humans such as the brain or testes are protected from inflammatory responses which may be damaging. These, known as immune‐privileged tissues, are additionally protected by physical barriers that normally prevent pathogen invasion [6, 7, 8]. Nonetheless, *T. gondii* has been amply shown to cross barriers, such as the blood–brain barrier (BBB) and the placental barrier [9]. However, the underlying mechanisms and consequences of *T. gondii* crossing the blood‐testes barrier (BTB) have not been explored. The BTB is a complex structure present in the seminiferous tubules that creates two testicular tissue compartments, the basal and the abluminal. The nurturing Sertoli cells build intercellular connections formed by tight junctions creating an isolated abluminal compartment [10]. *T. gondii* can retrogradely cross the BTB, from the epithelium to the seminiferous tubules, skipping the barrier of Sertoli cells. Ejaculate from *T. gondii‐*infected rats displayed the presence of viable tachyzoites [11]. In addition, these rats were able to infect mated females [11]. Interestingly, it was demonstrated that sperm from *T. gondii‐*infected mice can carry epigenetic factors that can contribute to the intergenerational inheritance of behavioural changes after pathogenic infection [12]. Even though the offspring from *T. gondii‐*infected mice were seronegative for *Toxoplasma*, they showed impaired spatial working memory, reduced mobility, and increased depression‐like phenotype. Interestingly, these factors were observed more prominently in male than in female offspring. This study also showed that *T. gondii* cysts can reside in murine testes for at least four weeks post‐infection. In addition, the presence of testicular *T. gondii* tissue cysts correlated with lower testes weight, sperm count and motility as well as increased abnormal sperm morphology [12]. One study found *T. gondii* cysts in the ejaculates of immunocompetent and latently infected human males. These findings were confirmed by the detection of an actively transcribed bradyzoite‐specific gene in these structures [13]. Female goats artificially inseminated with *T. gondii*‐spiked semen seroconverted, suggesting the potential for sexual transmission of *T. gondii* [14]. However, more experimental evidence, as well as studies focusing on additional species are needed to consolidate this route of transmission as well as the universality of these findings. Nonetheless, these data pose important considerations both to the current conception of toxoplasmosis as a disease, and importantly, to additional underappreciated mechanisms of horizontal transmission.

Although the sexual transmission of *T. gondii* has not been extensively studied in humans, a recent investigation by Hlaváčová *et al*. [15] examined its potential association with male infertility. The study found that 27% of men with semen pathologies and 22.3% of those with normozoospermia tested seropositive for *T. gondii*. Notably, up to 86.6% of *T. gondii*‐positive men exhibited semen abnormalities, including oligozoospermia, asthenozoospermia, and teratozoospermia [15]. Altogether these findings suggest a possible positive correlation between toxoplasmosis and sperm function in humans. Immunocompetent individuals who develop toxoplasmosis are mainly asymptomatic and the disease is seemingly resolved by the immune system. However, given the evidence that tachyzoites in male reproductive organs of animals can be infective in animal models and that sperm quality‐associated problems are more prevalent in seropositive individuals, it seems possible that *T. gondii* infection of either the human male testes, the epididymis or seminal glands could contribute to infertility. In this context, understanding the impact of *T. gondii* tachyzoites on human spermatozoa represents a fascinating, underexplored, intersection of parasitology and reproductive biology. A better understanding of the interaction of sperm and *T. gondii* tachyzoites could provide valuable insights into infectious disease‐associated mechanisms underlying male infertility, providing a window of opportunity for timely intervention. Here, we explore the interaction between fast‐replicating *T. gondii* tachyzoites – prevalent in the subclinical acute infection‐ and human sperm cells.

## Results

### *Toxoplasma gondii* tachyzoites infect murine testes and epididymis *in vivo*

**Fig. 1:**
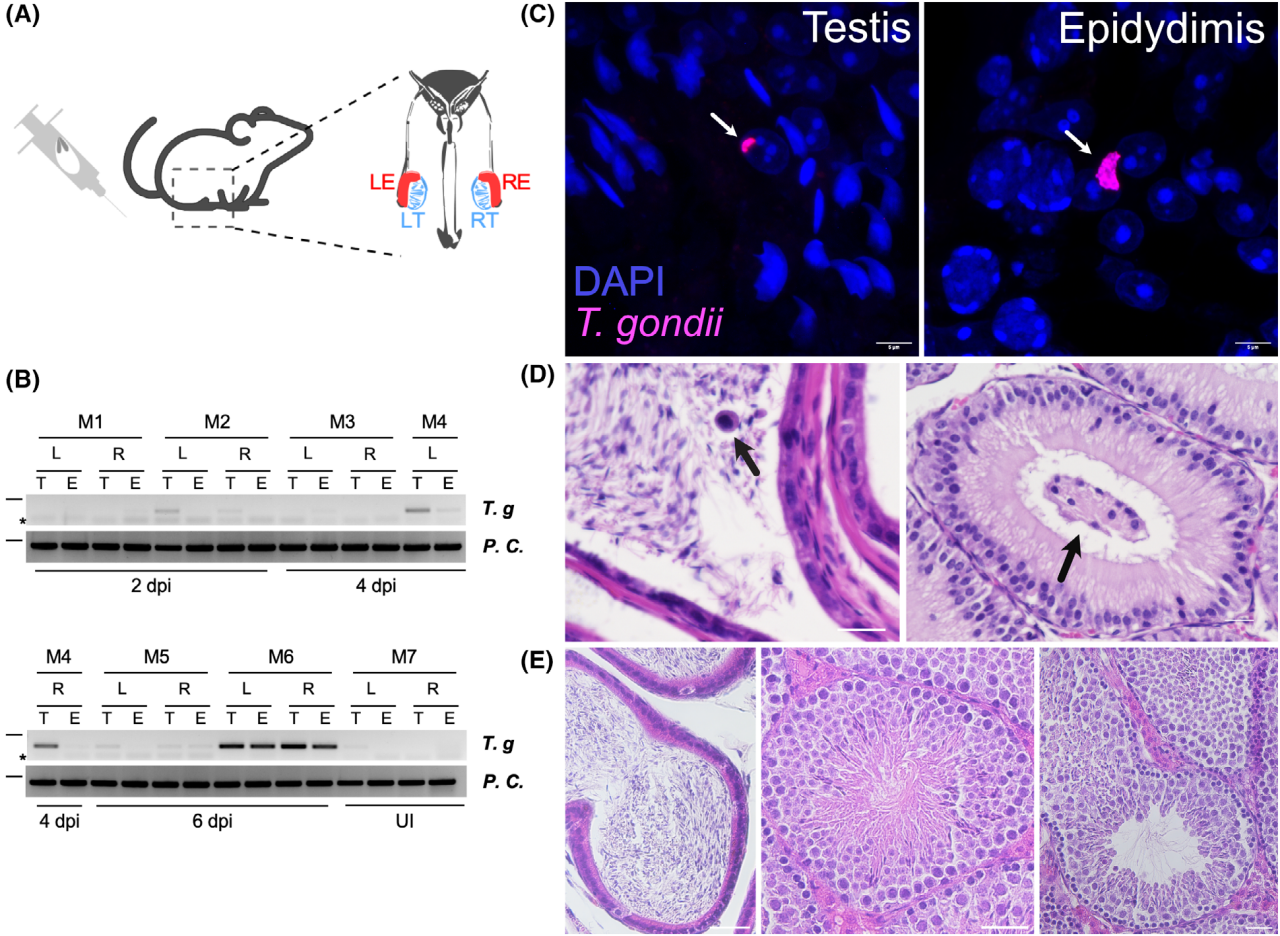
*Toxoplasma gondii* tachyzoites were detected in murine testes and epididymis after 2 and 6 days p.i. (A) Mice were infected intraperitoneally with *T. gondii* tachyzoites. After, 2, 4 and 6 days p.i. mice were sacrificed, and testes (T) and epididymis (E) were isolated. Both organs from the right (R) and the left (L) side of the body were separately processed for genomic DNA extraction. (B) *T. gondii*‐specific PCR analyses were used to detect tachyzoite DNA in testes (T) and epididymis (E) samples from 2, 4 and 6 days post infection (p.i). Positive and negative PCR controls: M1–M7 refers to distinct mice. UI, uninfected; *T.g*., *T. gondii* detection refers to *T. gondii* specific DNA amplification using the Tox5‐8 primer pair (see Materials and methods); Positive control refers to the amplification of a nuclear mammalian gene using the NPOCF primer set (see Materials and methods). The expected amplification products are 300 and 500 pb, respectively. Please note that dashes indicate the 500 bp mark of the molecular weight ladder. * denotes primer dimers. (C) Representative pictures of *T. gondii* tachyzoites (magenta) immunofluorescence detection in tissue sections. White arrows show *T. gondii* tachyzoite. DAPI: nuclear marker. Picture magnification: 120×. Scale bar: 5 μm. (D) Haematoxylin eosin‐staining in samples from T and E after 6 days p.i. The black arrow depicts the unusual localisation of non‐spermatozoa cells inside the seminal tube. The black arrow shows an immune cell inside the seminal tube. (E) Haematoxylin eosin from non‐infected animals is shown to compare with pictures in (D). Scale bar in (C): 5 μm and (E): 20 μm. Total mice used: 6.

To our knowledge, only two studies have unambiguously proven the presence of *T. gondii* tachyzoites in testes, in a dog and a rat, respectively [16, 17]. Therefore, to first verify that *T. gondii* indeed crosses the blood‐testes barrier and proliferates within cells of male reproductive organs in mice, we infected mice intraperitoneally with *T. gondii* tachyzoites (Me49 strain). testes and epididymis of these animals were screened for the presence of *T. gondii* after two, four and six days post‐infection (from here on referred to as p.i.) by PCR (Fig. 1A,B). Given that both structures are paired inside the body, we analysed independently the right‐ and left‐located tissue. We detected *T. gondii* DNA in as soon as two days p.i., in both testes and epididymis, independently of their anatomical position (Fig. 1B). At six days p.i., *T. gondii* DNA was detectable in all mice (Fig. 1B). We note, however, that large variations in the strength of PCR amplification were detected, presumably owed to individual variation. Since these results only proved the presence of parasite DNA in the testes and epididymis, we also tested the presence of tachyzoite in both organs by immunofluorescence assay. Serial microsections from testes and epididymis were immunostained with a *T. gondii* – a tachyzoite‐specific antibody that specifically recognises the parasite's surface proteins (Fig. 1C), which highlighted tachyzoites in both tissues after six days p.i., demonstrating that *T. gondii* indeed traverses the BTB, thereby infecting male reproductive organs in mice (Fig. 1C). Additionally, we studied the testes and epididymis structure in experimentally *T. gondii‐*infected mice to identify whether the infection induced any changes in the tissue structure. Our results show that six days p.i., immune cells (Fig. 1D – black arrow) and non‐spermatozoan cells were detected inside seminal tubes (Fig. 1D – yellow arrow). These features were not observed in similar sections in control mice (Fig. 1E). Altogether, these results confirm that, albeit in a small number of animals, *T. gondii* tachyzoites can likely infect male reproductive organs in as soon as two days p.i., and as we experimentally corroborated, six days p.i *in vivo*, modifying the structure of the testes and epididymis.

**Fig. 2:**
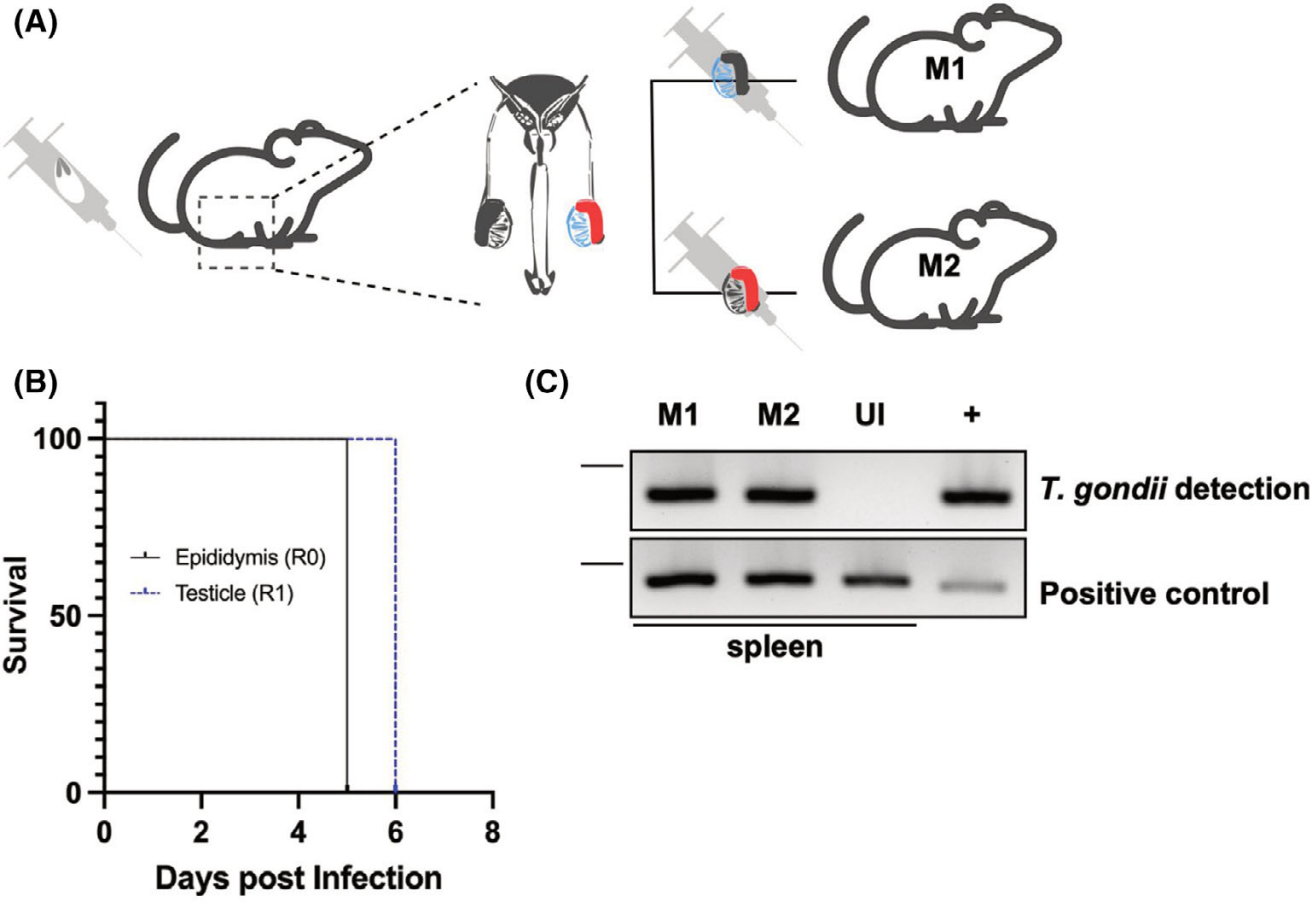
*Toxoplasma gondii* tachyzoites viability test in murine epididymis and testicles. (A) To prove that tachyzoite found in the epididymis and testicle tissue samples indeed remained infective, one B6 mouse was injected intraperitoneally with 1 × 10^3^ TgMe49 tachyzoites. After 6 days p.i., the mouse was sacrificed, and epididymis and testes were isolated. Tissue extracts corresponding to the testes (T) or the epididymis (E) were inoculated intraperitoneally into two additional B6 mice, respectively. Five days post‐inoculation (p.i.), the mouse infected via the epididymis (M2) was humanely sacrificed, followed by the mouse inoculated via the testes (M1) on the next day (6 days p.i.). During necropsies of the mice, spleens were collected and analysed for *T. gondii*‐specific DNA by PCR. Here, murine spleen samples revealed all positive. *T. gondii* detection refers to *T. gondii* specific DNA amplification using the Tox5‐8 primer pair (see Materials and methods); positive control refers to the amplification of a nuclear mammalian gene using the NPOCF primer set (see Materials and methods). The expected amplification products are of 300 and 500 pb, respectively. Please note that dashes indicate the 500 bp mark of the molecular weight ladder. + denotes amplification using tissue‐culture‐extracted *T. gondii* DNA; UI, uninfected mouse spleen. Total mice used = 4.

*Toxoplasma gondii* infection is characterised by rapid proliferation of tachyzoites within the infected tissue during acute toxoplasmosis. However, we were unable to observe replicating tachyzoites in our tissue sections. To unequivocally show that detected parasites were proliferative and remained infective we performed a viability test on both tissues. Mice were first infected with 1 × 10^3^ *Tg*ME49 tachyzoites by intraperitoneal injection (Fig. 2A). Six days p.i., mice were sacrificed, and both testes and epididymis were dissected. Tissue extracts were used to infect naive stem cells in two mice by intraperitoneal injection (Fig. 2A,B); five and six days p.i., both mice were sacrificed and the spleen was extracted, and *T. gondii*‐infection was confirmed by PCR (Fig. 2C). Altogether these results suggest that detected tachyzoites within testes and epididymis remained viable and infective. Quantification of the parasite load in these tissues via quantitative PCR showed that, on average, each testes and epididymis (right and left) was colonised by approximately 1.3 × 10^2^ parasites, representing 12% of the initial inoculum per tissue (Fig. S1). Given this, it is highly unlikely that such a distribution—close to 50% of the total initial inoculum—would occur randomly in these tissues upon injection. In addition, given the celerity with which the mice deteriorated, we hypothesise that the number of parasites present in the injected tissue was high, arguing that tachyzoites are indeed able to proliferate within the testes and epididymis.

### Direct attachment of *Toxoplasma gondii* affects human spermatozoan structure and morphology

**Fig. 3:**
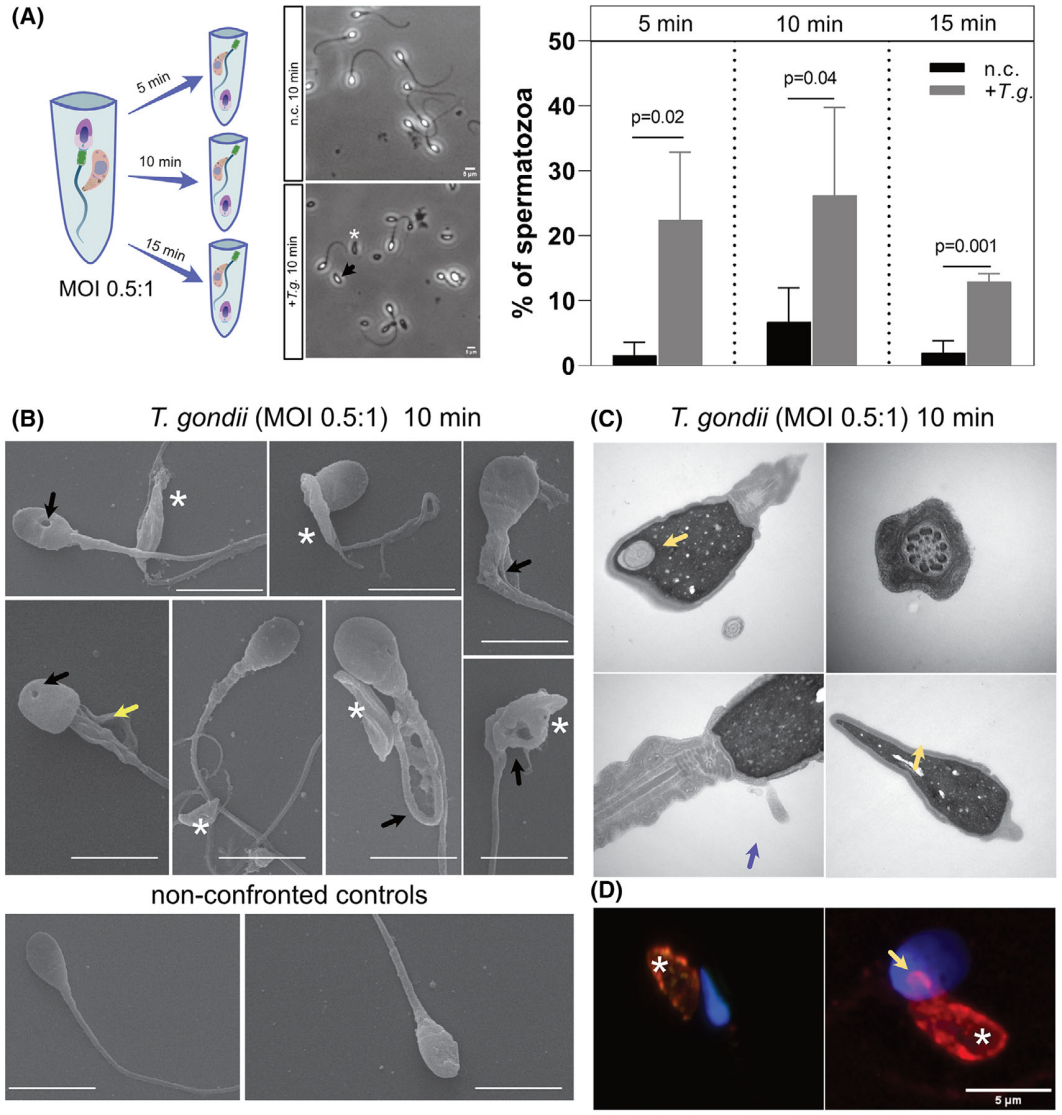
Human spermatozoa confronted with *Toxoplasma gondii* tachyzoites lead to sperm integrity defects. (A) Spermatozoa from three human donors (*n* = 3) were confronted with *T. gondii* tachyzoites (MOI 1 : 0.5) for 5, 10 and 15 min. At each time, the total number of spermatozoan heads was counted and normalised with the total number of cells in the field of view. The plots show the percentage of spermatozoan heads in each sample. Bars represent the percentage of three donors ± SD. Representative images are shown at 10 min p.c. displaying *T. gondii* tachyzoites (asterisks) and spermatozoan heads (black arrow). (B) SEM pictures at 10 min p.c. to analyse spermatozoan morphology (*n* = 2). Asterisks depicted parasite localisation and arrows, defects in spermatozoan tails or heads. (C) TEM pictures show spermatozoan head holes (yellow arrows) and plasma membrane detachment region (blue arrow). (D) Immunofluorescence shows that *T. gondii* tachyzoites (asterisks) are attached to the spermatozoan heads by the parasite lateral region and eventually extruding the conoid for infection attempts. Scale bar in (A) and (D): 5 μm; (B): 2 μm.

The confirmation that *T. gondii* was able to infect testes and epididymis, and parasites are viable led us to explore the effects of tachyzoite contact either with germinal cells during spermiogenesis or with mature spermatozoa. To observe this preliminarily, we optimised *in vitro* experiments involving the confrontation of human spermatozoa from healthy donors with viable *T. gondii* tachyzoites. Healthy sperm donor samples (*n* = 3) were subjected to ‘swim‐up’ assays, which allowed the purification of motile and healthy spermatozoa only, from each ejaculate. These were then confronted with vital and motile *T. gondii* tachyzoites (at a multiplicity of infection, MOI of 0.5 : 1) at time intervals ranging from 5–15 min. Results show that even after only 5 min post confrontation (from here on referred to as p.c.), 22.4% of the counted spermatozoan were headless when compared to control sperm samples (1.6%) (Fig. 3A). Similarly, 10‐ and 15‐min confrontations led to an increase in spermatozoa lacking heads of four and eight folds, respectively (Fig. 3A). To analyse this tachyzoite‐derived effect on exposed human sperm in more detail, samples were submitted to scanning electron microscopy (SEM) analysis. *T. gondii* tachyzoites were observed in juxtaposition to spermatozoan's tails or heads (Fig. 3B – asterisks). Notably, several spermatozoa displayed twisted and rolled up or right angle tails. Strikingly, we observed a multitude of free spermatozoan heads. Some of these heads appeared to be void of content, but additional structural aberrations including holes could be observed (Fig. 3B – black arrow). Further insights by transmission electron microscopy (TEM) showed that holes in sperm heads were accompanied by focal plasma membrane detachments (Fig. 3C – yellow and blue arrows, respectively). Interestingly, immunodetection of *T. gondii* tachyzoites demonstrated that the parasite contacted the spermatozoan head by its anterior end, where all its invasion machinery resides, suggesting an attempt to invade male gametocytes (Fig. 3D – yellow arrow).

**Fig. 4:**
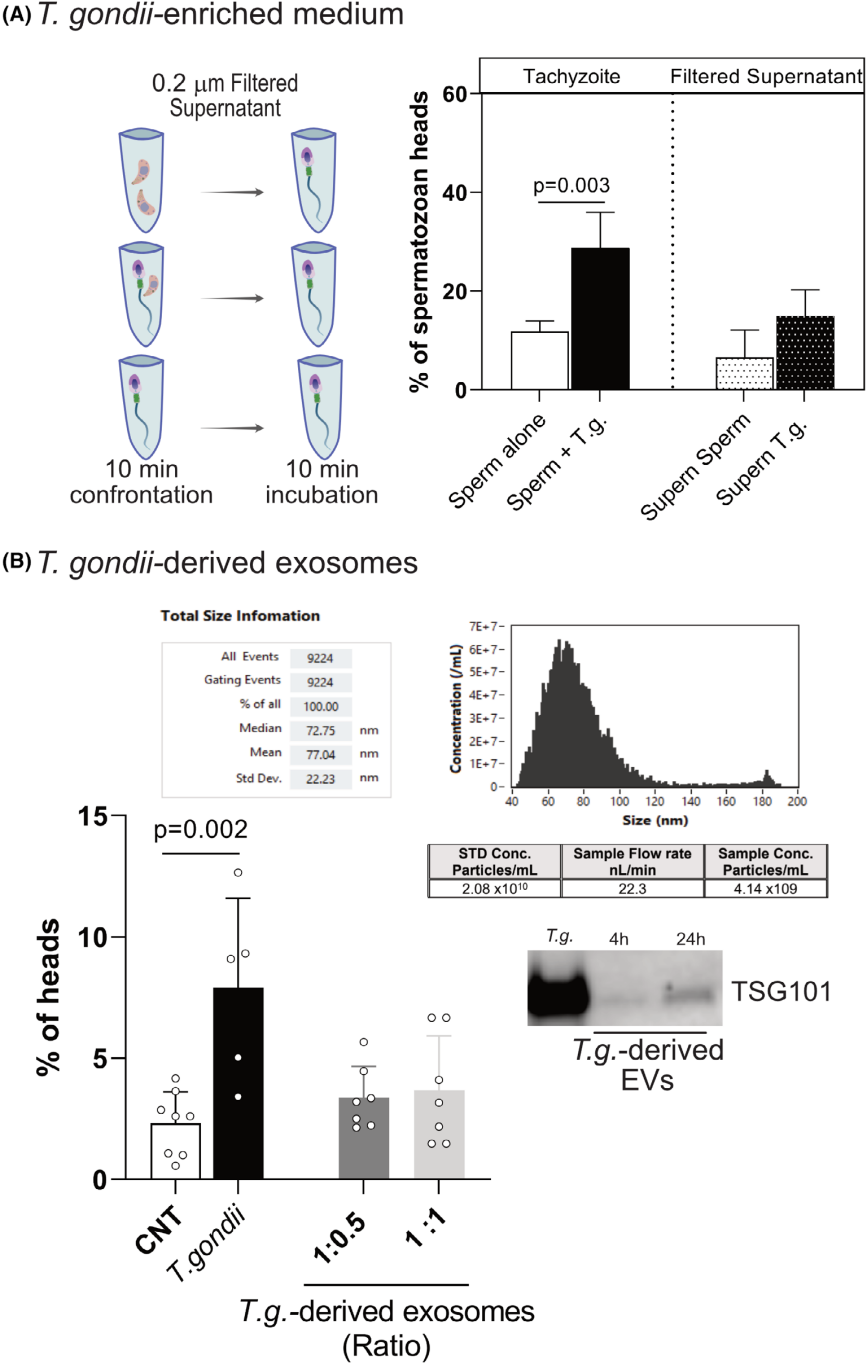
Treatment of sperm with infection‐conditioned medium or parasite EVs fails to induce spermatozoa beheading. (A) Spermatozoa from eight human donors (*n* = 8) were confronted with sperm previously confronted with *Toxoplasma gondii*‐infection conditioned medium for 10 min (black dotted bar). As a control, sperm‐enriched supernatant (white dotted bar) was used as well as a confrontation directly with tachyzoites (+*T.g*. Black bar). Thereafter, the total number of detached spermatozoan heads was counted and normalised to the total number of cells in the field of view. The plots show the percentage of spermatozoan heads in each sample. Bars represent the percentage of eight donors ± SD. (B) The same experiment was performed but adding *T. gondii*‐derived exosomes at a 1 : 0.5 or 1 : 1 ratio to sperm. Bars represent the percentage of nine donors ± SD.

It is well established that *T. gondii* host cell invasion is a hierarchical process, where the content of secretory organelles is sequentially released into the host cell membrane, enabling attachment and host cell modification. In addition, *T. gondii* releases soluble factors that modify distant cells, altering, for example, their progression through the cell cycle [18]. In this context, we wondered whether the effect of *T. gondii* tachyzoite on the spermatozoan head or tails was related either to immediate tachyzoite contact with spermatozoa or mediated by secreted molecules/proteins. To discern between these alternatives, we isolated supernatant by filtering the non‐confronted (n.c.) sperm, *T.g*.‐confronted sperm and tachyzoites‐alone for 10 min. These supernatants were co‐incubated with n.c. spermatozoan during 10 min and after fixation the number of headless spermatozoa was counted (Fig. 4A). Overall, *T.g*.‐supernatant treatment had no significant effect on the number of headless spermatozoa (15%), while cells confronted directly with *T. gondii* tachyzoites increased the number of detached heads in 28.8% (Fig. 4A). Thereby, a direct effect by soluble parasite effector molecules being secreted into the medium was excluded. To further explore this phenomenon, we confronted spermatozoa with *T. gondii*‐derived extracellular vesicles (EVs) (i.e. purified exosomes), testing two ratios of sperm : exosomes, 1 : 0.5 and 1 : 1. The results show again no effect of *T. gondii‐*derived exosomes in the number of headless spermatozoa, excluding any role of EVs (Fig. 4B).

Taken together, our results show for the first time that head loss after *T. gondii* tachyzoites confrontation is not mediated by soluble factors released into the medium. Instead, the generation of headless spermatozoa requires parasite presence and/or direct contact.

### *Toxoplasma gondii* decreases human sperm mitochondrial membrane potential without altering reactive oxygen species (ROS) prodution

**Fig. 5:**
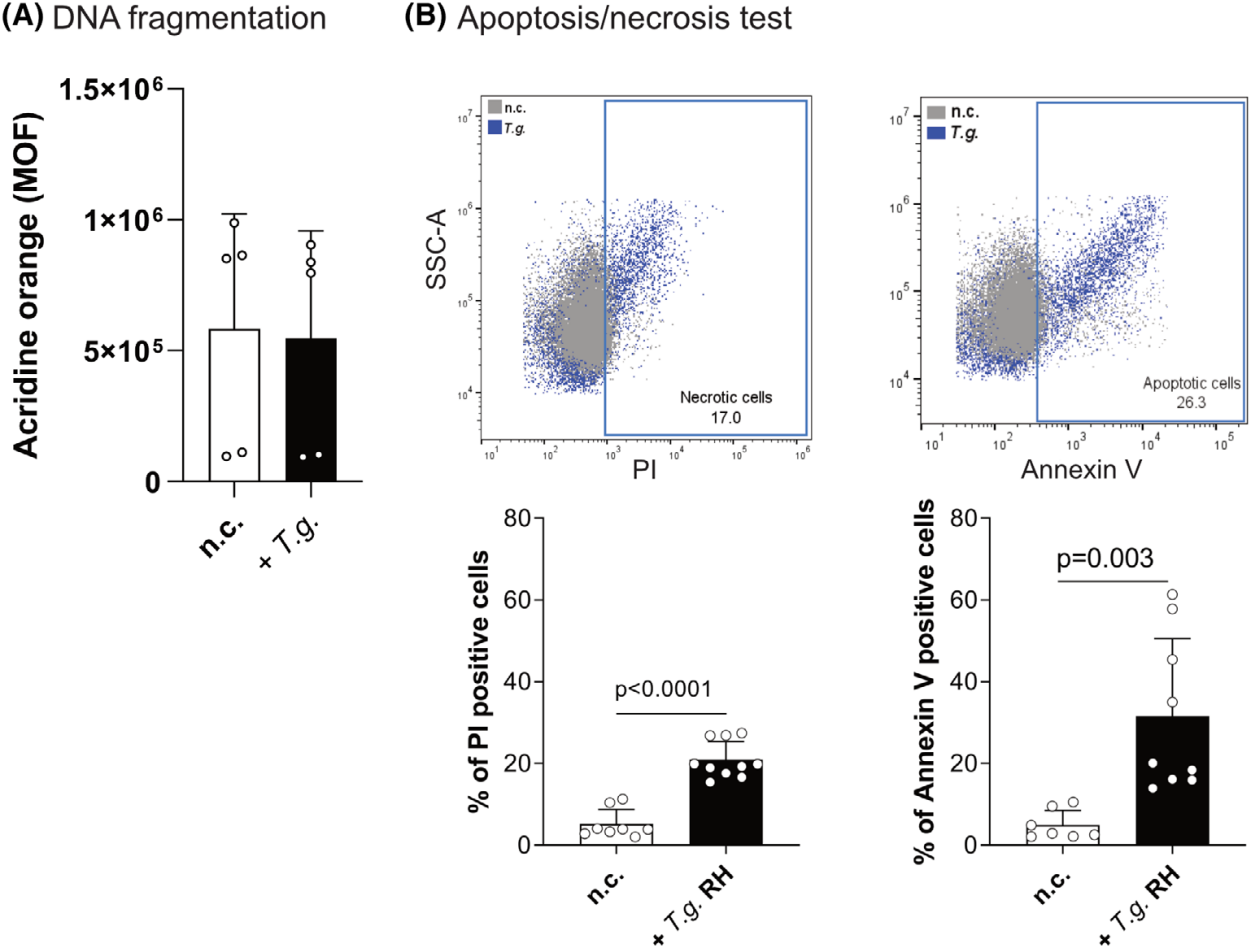
Sperm confronted with *Toxoplasma gondii* increased apoptosis and necrosis without changes in DNA integrity. (A) Study of the sperm DNA fragmentation after *T. gondii* confrontation (*n* = 4). Single and double DNA strands were stained with acridine orange. Bars represent the percentage of four donors ± SD. (B) Analysis of the apoptosis/necrosis induced by *T. gondii* after 10 min of confrontation with human spermatozoan. Bars represent the percentage of cells positive to Anne xin V or PI of nine human donors ± SD.

To delve further into the mechanisms of sperm decapitation by *T. gondii*, we assayed sperm DNA integrity. For this, cells were stained with acridine orange which displays two different wavelength signals depending on whether it is attached to single or double‐stranded DNA. Our findings show no increase in single‐stranded‐DNA after 10 min of *T. gondii* tachyzoite confrontation, suggesting that no DNA fragmentation was induced by this parasite stage (Fig. 5A and Fig. S2). Subsequently, we aimed to analyse whether cells undergo apoptosis and/or necrosis following *T. gondii* confrontation. Both processes can be quantified using FACS with simultaneous Annexin V and propidium iodide (PI) staining. This method enables the detection and differentiation of apoptotic and necrotic cells within the same sample. To interpret the results more straightforwardly, both channels were analysed independently. We quantified the percentage of cells undergoing either apoptosis or necrosis 10 min p.c., observing that both apoptosis (31.6%) and necrosis (28.9%) increased compared to the negative control (n.c.) sperm cells (~ 5.1%, Fig. 5B). Although the percentages of apoptosis and necrosis are similar, apoptosis exhibited two distinct populations (16.9% and 52.2%), suggesting that *T. gondii* tachyzoites primarily trigger apoptosis initially, and subsequently induce necrosis, thereby killing human spermatozoa during the confrontation.

**Fig. 6:**
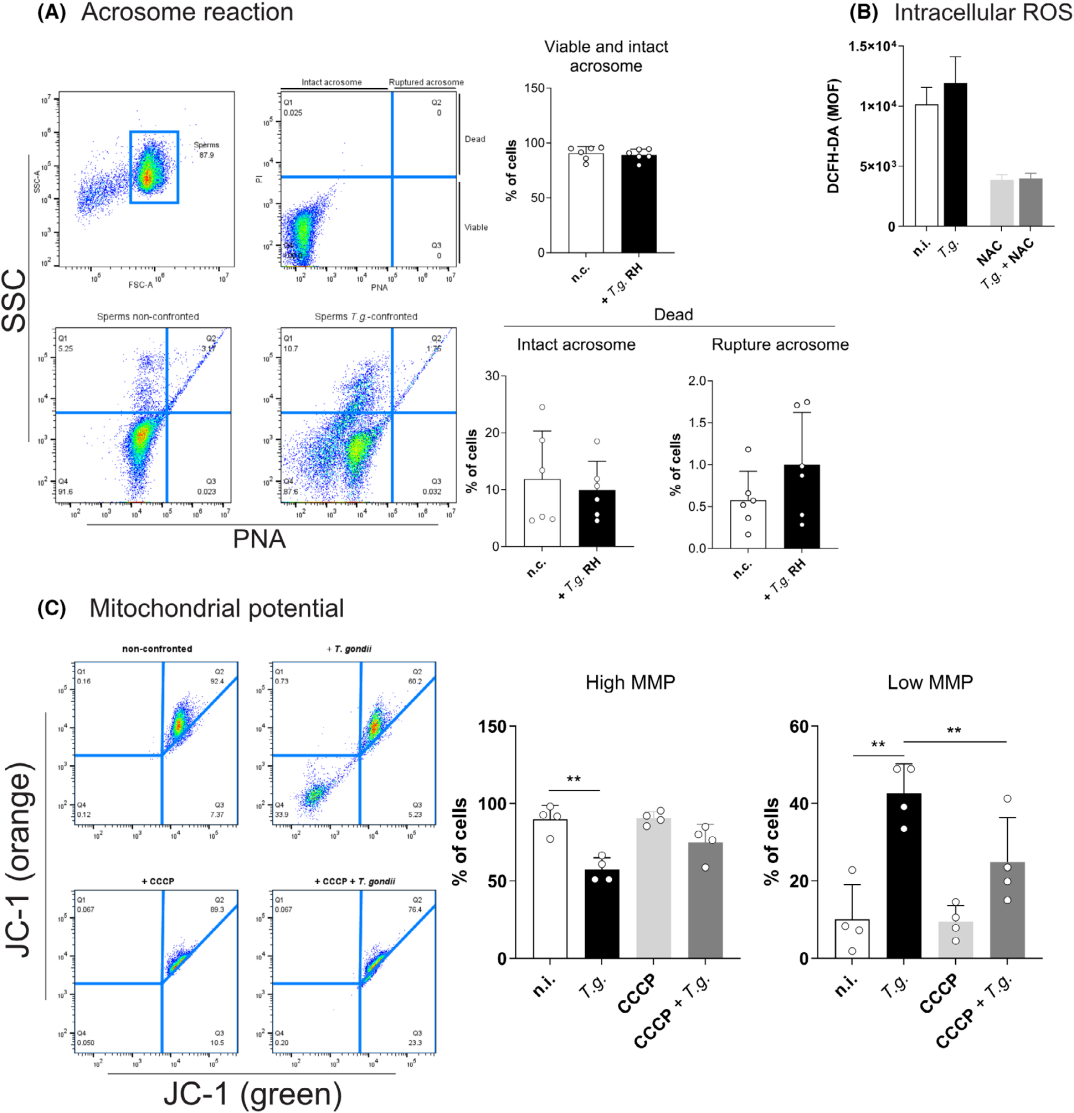
Exposure to tachyzoites does not affect sperm acrosome reactions but decreases the mitochondrial membrane potential in human spermatozoa. (A) Acrosome reaction was analysed by the PNA lectin stain in spermatozoa. Samples were incubated for 10 min with *Toxoplasma gondii* tachyzoites and read by FACS. Bars represent the percentage of cells of five donors ± SD. (B) Quantification of mitochondria membrane potential (MMP) by pre‐incubation of sperm cells with JC‐1 probe. Spermatozoans were then confronted with *T. gondii* tachyzoites for 10 min and the red or green JC1 signal was analysed by FACS. Bars represent the percentage of cells positive for red (high potential) or green (low potential) of six donors ± SD. (C) Quantification of intracellular ROS by FACS. Human sperm were confronted with *T. gondii* tachyzoites for 10 min, and then the DCFH fluorescence was read. Bars represent the percentage of cells positive to DCFH of six donors ± SD. As a negative control, sperm were pre‐incubated with ROS inhibitor, NAC. **P ≤ 0.01.

The acrosome is a membranous organelle located over the anterior part of the sperm nucleus. The so‐called acrosome reaction is an important process in male fertility. This reaction triggers a calcium flux‐induced fusion of the acrosomal membrane with the sperm's plasma membrane and defects in this process may affect the ability of sperm to fuse with the zona pellucida of oocytes. We analysed the effect of *T. gondii* confrontation on the acrosome reaction, observing that neither viable nor dead spermatozoa increased acrosome rupture after being incubated with *T. gondii* for 10 min (Fig. 6A).

Spermatozoa are highly motile cells that rely upon mitochondrial ATP‐derived energy for their continued motility [19]. Sperm mitochondria not only participate in spermatozoa motility but are critical for hyperactivation, capacitation, acrosome reaction and fertilisation [20]. Mitochondria are also a major source of ROS, and human spermatozoa are particularly sensitive to ROS in semen. We hypothesise that the beheading phenotype could be mediated by a response triggered by oxidative stress (OS) resulting in ROS production. To test this, a DCFH‐DA cell‐permeable probe was used for detecting intracellular oxidative stress. DCFH carboxylate anion is retained within the cell. Intracellular oxidation of the probe to DCFH reacts with various ROS results in the formation of the highly fluorescent product that was quantified by FACS. Intracellular ROS quantification following 10 min of *T. gondii* confrontation showed no changes in comparison to the n.c. samples which suggest that *T. gondii* did not modulate ROS concentration (Fig. 6B). As an assay control, cells were pre‐incubated with a potent ROS inhibitor, NAC. Given the mitochondria's prominent role in sperm physiology, we explored mitochondrial membrane potential (MMP) by incubating cells with the JC‐1 probe (Fig. 6C). The MMP represents the voltage difference between the mitochondrial matrix and the cytosol. The method used in this experiment involves JC‐1 dye, which is a widely recognised probe for assessing MMP. In cells with high MMP, JC‐1 forms J‐aggregates that emit orange fluorescence, while in cells with low MMP, the dye remains in its monomeric form, emitting green fluorescence (Fig. 6C – FACS flow chart). As an assay control, cells were treated with the inhibitor of mitochondrial oxidative phosphorylation, CCCP (carbonyl cyanide m‐chlorophenyl hydrazone). CCCP disrupts the mitochondrial inner membrane potential by uncoupling oxidative phosphorylation, leading to a dissipation of the electrochemical gradient. Our findings show a significant decrease in MMP 10 min p.c. This result is supported by both decreased percentages of cells with a high MMP and increased percentages of cells with low MMP after *T. gondii* confrontation (Fig. 6C). Even though this result supports that *T. gondii* might affect sperm mitochondrial activity, we considered that this data has to be correlated with the high percentage of cells that undergo necrosis (Fig. 5B). Therefore, the real effect of *T. gondii* tachyzoites confrontation on mitochondrial sperm must be further studied.

## Discussion

Herein we show, for the first time, that the direct contact between mature human spermatozoa and *T. gondii* tachyzoites triggers ultrastructural changes in the former, leading to an increase in the percentage of headless sperm cells. Given that chronic toxoplasmosis prevalence ranges between 1% and 100% in the global human population, depending on geographical location [2], and the presence of cysts in human testes has been documented [5], re‐activation of these cysts into fast‐replicating tachyzoites could have an underappreciated impact on male fertility.

Our results show that contact between *T. gondii* tachyzoites and sperm leads to an increased loss of spermatozoan heads. The generation of headless spermatozoa has only been described before in the context of an underlying genetic condition known as acephalic spermatozoa (ASS), which is one of the most severe spermatogenic failures of all infertility causes in men [21]. To date, only the disturbance of the sperm head‐tail coupling apparatus (HTCA), caused by mutations in genes that conform to the HTCA complex has been mechanistically associated with ASS [22, 23]. Herein, we identify that human sperm lose their heads upon direct contact with tachyzoites, highlighting that neither *T. gondii‐*enriched medium nor *T. gondii*‐derived EVs had an adverse effect on exposed gametocytes.

The fact that we detect viable *T. gondii* tachyzoites both in the testes and the epididymis *in vivo* strongly argues that the parasite can cross the BTB and come in contact with sperm. In a physiological context, physical contact of this parasite with germ cells might occur during spermiogenesis inside the testes or the epididymis. Prior studies in mice had already demonstrated that *T. gondii‐*infected mice displayed problems in spermatozoan morphology and suffered a decrease in their total number, even four weeks p.i. [12]. In line with this, histopathologic studies of the reproductive system in male sheep experimentally infected with *T. gondii* showed a pro‐inflammatory process in the prostate gland and seminal vesicles. These results were also linked to an increased risk of parasite‐exposed individuals to develop prostate cancer as a consequence of chronic inflammation [24]. In order to avoid human prenatally acquired (congenital) toxoplasmosis, exposure to *T. gondii* is routinely screened serologically in pregnant women in several countries [25]. However, men are rarely analysed for the presence of *T. gondii*‐specific antibodies. Given that acute toxoplasmosis is rarely symptomatic in immunocompetent individuals, infections at any stage of male sexual development go mostly unnoticed by urologists or andrologists. Hence, the impact of human toxoplasmosis on spermiogenesis through sexual development is completely unknown. In addition, it is well known that *T. gondii* can remain dormant inside the mammalian host, and in fact, cysts with viable bradyzoites have been observed in human semen and biopsies of testes [5, 13]. A limitation of the present work is that all experiments were performed with mature human sperm. However, our results pose interesting questions to be tackled in the future, demanding more investigation into this neglected topic of human and animal andrology. The detection of viable *T. gondii* tachyzoites in both the testes and epididymis *in vivo* provides strong evidence that the parasite is capable of crossing the BTB and coming into contact with sperm.

Tachyzoites incubated with sperm cells showed twisted contortion, gliding motility as well as conoid protrusion, all early hallmarks of host cell invasion. Morphological alterations observed in spermatozoa as early as 5 min p.c. suggest that the parasite tried to invade the sperm cell as fast as it is described for somatic cells [26]. It is well known that polyxenous *T. gondii* can infect almost any nucleated cell of warm‐blooded mammals. However, the invasive capacity of *T. gondii* tachyzoites in germ cells has not been assayed before. Unlike most common experimental systems, which make use of somatic diploid cells – usually containing a vast cytosol for housing the parasite – sperm are highly motile haploid cells, with a headspace mostly occupied by the nucleus, the acrosome, the sub‐acrosomal space, and a tail. Therefore, it is possible that though sperm cells have a plasma membrane that could display the universal host cell receptor used by the parasite to attach itself, the formation of a parasitophorous vacuole (PV) might be limited by the minute cytosolic space available in a human sperm cell. Further experiments are needed to address this hypothesis.

Due to the critical role of mitochondria in sperm‐related function, any alteration in mitochondrial activity can cause oxidative stress leading to male infertility [27, 28]. SEM images from *T. gondii‐*confronted spermatozoa revealed that some mitochondrial regions were emptied. Upon determination of MMP, we demonstrated that it decreased 10 min p.c., indicating a rapid collapse of the membrane potential upon parasite encounter. The loss of MMP might be accompanied by a significant drop in ATP levels and a breakdown of the outer membrane. Loss of membrane potential usually precedes the release of mitochondrial pro‐apoptotic factors, i.e. cytochrome C, the activation of caspases and finally the activation of apoptosis, a phenomenon also undergone by *T. gondii*‐confronted sperm cells. ROS molecules have been associated with the loss of membrane potential; however, we detected no changes in intracellular ROS 10 min p.c. These results rather argue for a ROS‐independent sperm cell death mechanism but need further investigation for a conclusion.

An additional implication of the current results is that not only tachyzoites of *T. gondii* might affect male fertility patients with acute toxoplasmosis but also during chronic infections; bradyzoites within tissue cysts (as shown previously to exist both in humans and other animals), could pose a risk for sexual and congenital transmission. In human *T. gondii* infections, a sexual route of transmission was recently proposed by Ullmann *et al.* [29] based not only on the presence of this parasite in human/animal seminal fluid but also on the higher prevalences of toxoplasmosis in men and women practising fellatio [15]. Nevertheless, these assumptions are based on correlations of reported data; future experimentation is needed to verify this hypothesis.

Finally, we would like to address the physiological relevance of the results shown in the current work. Given that primary *T. gondii* infections typically involve the tachyzoite stage, we hypothesise that spermatozoa could come into direct contact with tachyzoites if sexual transmission, as previously described, indeed occurs. However, this route of transmission is not well studied, and we prefer to wait for more evidence before firmly suggesting such contact between the two cell types. Nonetheless, our current results in mice infected with *T. gondii* tachyzoites revealed that tachyzoites reached the testes and epididymis as early as 2–6 days p.i. They not only infected the tissues but were also observed in the lumen of seminiferous tubules, where mature sperm cells are released before ejaculation. Based on these findings, we can suggest that *T. gondii* tachyzoites may indeed come into direct contact with mature sperm cells within the testes. While it is true that the doses of tachyzoites used to infect these mice were lower than those used in the *in vitro* confrontation experiments, we believe the extended duration of exposure between the sperm cells and parasites within the testes compensates for the lower parasite‐to‐cell ratio. Overall, current data raise important questions about the potential impact of the zoonotic‐relevant *T. gondii* on male fertility, with implications for both public health and animal welfare. Moreover, further detailed research on this matter is needed to evaluate the importance of educating humans on preventive measures to reduce the risk of human toxoplasmosis transmission and its potentially unforeseen consequences on human and animal reproductive health.

## Materials and methods

### Animal experiments

To evaluate the viability of the parasites in the epididymal and testicular tissues, 6–8‐week‐old male C57BL/6 mice were infected with 1 × 10^3^ TgME49 *T. gondii* tachyzoites via intraperitoneal injection. Six days after the infection, the mice were sacrificed and tissue samples from the epididymis and testicles were obtained. Tissue samples from two mice were fixed in a 10% buffered formalin solution and further used for histological studies. Both epididymal and testicular tissues from *T. gondii‐*infected mice were inoculated intraperitoneally into two additional C57BL/6 mice. Necropsies of these animals were performed after two and six days p.i. The spleen was collected and, following DNA isolation, samples were analysed by PCR to confirm *T. gondii* infection. All experiments involving animals were done following pre‐approved protocols by the Institutional ethic committee (CEUA # 023‐22) at the Institut Pasteur de Montevideo, Montevideo, Uruguay. The institutional ethics committee is supervised at the national level by the National Commission of Animal Experimentation (CNEA) and the Honorary Commission of Animal Experimentation (CHEA). All animal experiments were performed following national regulations, imposed by the national law of animal welfare (Law 18611 ‘PROCEDIMIENTOS PARA LA UTILIZACIÓN DE ANIMALES EN ACTIVIDADES DE EXPERIMENTACIÓN, DOCENCIA E INVESTIGACIÓN CIENTÍFICA.’). Animals were bred in‐house at the animal housing facility of Institut Pasteur de Montevideo and maintained herein in controlled light and temperature conditions. A maximum of six animals were housed per cage and food and water were provided *ad libitum*. Bedding and environmental enrichment props were changed weekly. Parasite inoculation and mice euthanasia were performed as specified in the approved animal‐handling protocol (#023‐22), following previously established protocols optimized by our laboratory (see Cabrera *et al.* [30] for reference to prior published work).

### Human samples experiments

All described experiments on human semen samples were done according to ethical vote number AZ: 32/11, approved by the Justus Liebig University Giessen's institutional safety commission and following the standards set by the Declaration of Helsinki. All donors received an explanation about all experiments that will be conducted with their samples, previous the donation. All experiments were conducted with the informed written consent of each participant. All samples were collected in the Clinic for Urology, Justus‐Liebig‐University Giessen, Germany (Prof Dr med Florian ME. Wagenlehner) by a technician. They were delivered to us with a code that allowed them to remain anonymous. Samples were collected between July 2023 and July 2024.

### Parasite maintenance

*Toxoplasma gondii* tachyzoites (RH and Me49 strain) were maintained by serial passages in primary HFF cells (ATCC, Manassas, VA, USA, maximum passage: 10) as previously described in Ref. [31]. Freshly released *T. gondii* tachyzoites were harvested from HFF supernatants, pelleted (400 ***g***, 12 min), counted in a Neubauer chamber and resuspended in the corresponding medium for each host cell type. All experiments were performed at an MOI of 1 : 1 or 1 : 0.5 (spermatozoa : parasites) unless otherwise stated. To better discriminate sperm heads and tachyzoites, quantification of headless spermatozoan was performed using *T. gondii* RH tachyzoites expressing mCherry.

### PCR‐based detection of *Toxoplasma gondii* DNA in epididymis and testes tissues

Standardised diagnostic PCR was carried out following the protocol described by (Homan *et al*. [32]; Michael *et al*. [33]; Schares *et al*. [34]; Yamage *et al*. [35]), using a set of control primers (extraction control; NPOCF: 5′ GCATCCTTGAGTGTGAAGAGAA 3′ and NPOCR: 5′ TGCCTCATAAACTCACTGAACC 3′; which amplifies a 300 bp product in all samples arising from mammalian tissue or tissue culture listed in Figs 1 and 2 as ‘positive control’ amplification product) and a pair specific to *T. gondii*; TOX8 5′ CCCAGCTGCGTCTGTCGGGAT 3′ and TOX5 5′ CGCTGCAGACACAGTGCATCTGGATT 3′ (which amplifies a 500 bp product, listed in Figs 1 and 2 as ‘*T. gondii* detection’ amplification product). In short, amplification was carried out using MangoMix (Bioline, Cincinnati, OH, USA) following the manufacturer's recommendations. The amplification cycle consisted of an initial denaturation step at 94 °C for 5 min, followed by 35 cycles of 94 °C for 30 s, 55 °C (for NPOCF primer pair)/60 °C (for TOX5‐8 primer pair) for 1 min and 72 °C for 30 s. A final extension round was done for 10 min at 72 °C.

### *T. gondii* detection in tissue samples via immunohistochemistry and immunofluorescence

Testes were dissected from C57BL/6‐strain male mice infected with *Tg*ME49 tachyzoites and fixed in 10% formalin solution or Bouin's solution. Afterwards, they were embedded in paraffin following a standard protocol. Paraffin‐embedded tissues were sectioned at 4 μm thickness. For every 10th histological section, samples were submitted to a standard Haematoxylin/Eosin staining and observed in a BZ‐X800 microscope (Keyence Corporation, Itasca, IL, USA). Brightfield images were obtained using 20× magnification.

Immunofluorescence detection of *T. gondii* tachyzoites was performed every 10 microslides, similarly to the HE stains. First, paraffin‐embedded sections were deparaffinised by ethanol (75%, 80%, 90%) and xylol (100%) incubation, and incubated in warm 10 mm sodium citrate, pH 6 during 10–20 s. Samples were washed and blocked with PBS 1×‐3% BSA‐0.3% Triton X‐100 for 1 h at RT. Then, the samples were incubated with primary antibodies (anti‐*T. gondii* 1/100, PA1‐7256; Thermo Fisher Scientific, Dreieich, Germany) overnight at 4 °C in a humid chamber. Sections were washed with PBS and probed with a secondary antibody (Alexa Fluor 594 1 : 500, anti‐goat; Thermo Fisher Scientific) sequentially. Nuclei counterstaining was done with DAPI in combination with the mounting medium (Fluoromount G‐DAPI; Thermo Fisher Scientific).

### Semen samples and related analyses

Semen samples were obtained from 30 healthy volunteer donors (aged between 20 and 38 years). Donors were informed of the study protocol and their privacy was guaranteed. All participants signed written informed consent forms before participating in this research, agreeing that their semen samples would be exclusively used for scientific purposes. The study was conducted following the Ethic Committee Justus Liebig University Giessen, Germany (AZ:32/11).

Semen samples were collected by masturbation after 3–5 days of abstinence. After 30 min of liquefaction, all semen samples were examined according to the WHO 6th Edition Laboratory Manual [36]. Motile spermatozoan were obtained by the swim‐up technique. Briefly, conical tubes containing 0.35 mL of semen sample were supplemented with 0.7 mL of warm Sperm Preparation Medium (10700060; Origio, Trumbull, CT, USA) at the bottom of each tube. Tubes were then kept inclined at an angle of 45° and incubated at 37 °C for 60 min. The uppermost 0.5 mL of the medium, containing highly motile spermatozoa, was collected. The sperm number was calculated using a Neubauer chamber using a bright‐field microscope (Olympus, Merck KGaA, Darmstadt, Germany).

### Transmission electron microscopy

Transmission electron microscopy analysis was performed in 2 × 10^7^ human sperm confronted with 10 × 10^6^ *T. gondii* tachyzoites after 10 min of co‐incubation. Samples were fixed in 1.5% formaldehyde and 1.5% glutaraldehyde (Merck KGaA) in 0.15 m HEPES buffer (Merck KGaA). Samples were processed following the same protocol described previously in Ref. [37]. Briefly, samples were centrifuged at 1000 ***g*** for 10 min and the pellet was washed and postfixed in a buffer containing 1% osmium tetroxide. After several steps of distilled water washing, the samples were incubated overnight in 2% aqueous uranyl acetate at 4 °C, dehydrated in ethanol and embedded in Agar 100 resin (Agar Scientific, Essex, UK). Ultrathin sections of the cured blocks were mounted on formvar‐coated grids and stained with uranyl acetate and Reynolds lead citrate. Ultrathin sections were analysed in a transmission electron microscope (EM 902N; Zeiss, Oberkochen, Germany), equipped with a slow‐scan 2K CCD camera (TRS, Tröndle, Moorenweis, Germany).

### Scanning electron microscopy

Scanning electron microscopic analysis was performed in 20 × 10^6^ human sperm, confronted during 10 min with 10 × 10^6^ *T. gondii* tachyzoites on 10 mm diameter glass coverslips (Thermo Fisher Scientific). After incubation, cells were fixed in 2.5% glutaraldehyde (Merck KGaA), post‐fixed in 1% osmium tetroxide (Merck KGaA), washed in distilled water, dehydrated, critical point dried by CO_2_ treatment, and sputtered with gold. Finally, all samples were analysed via scanning electron microscopy (Philips XL30, Eindhoven, the Netherlands) at the Institute of Anatomy and Cell Biology, Justus Liebig University Giessen, Germany.

### Isolation of *Toxoplasma gondii*‐derived extracellular vesicles

*Toxoplasma gondii* tachyzoites (1 × 10^8^) were collected by scrap and filtered with a 3 μm filter (Millipore, Billerica, MA, USA) and centrifuged at 3000 ***g*** for 10 min. The pellet was washed out by centrifugation and finally resuspended in 12 mL of complete medium (as described for parasite maintenance) with bovine foetal serum depleted of EVs (BFS; Sigma‐Aldrich, Darmstadt, Germany). The suspension was incubated for 24 h and thereafter centrifuged at 3000 ***g*** for 10 min to remove parasites and cell debris. The supernatant was filtered with a 0.45 μm syringe filter (Sarstedt, Nümbrecht, Germany) to remove potential membrane fragments from lysed cells and large vesicles (> 500 nm). Then, it was ultracentrifuged at 10000 ***g*** for 30 min, recovering the supernatant with the larger vesicles. The sample's volume was reduced by using a 100 kDa Amicon Ultra tube (Merck KGaA) and centrifuged at 3000 ***g*** per 8 min. After this centrifugation, the extracellular vesicles (EV) were separated by size exclusion chromatography (qEV original 70‐nm columns; IZON Science Ltd., Lyon, France) following the manufacturer's instructions. Finally, the EV fractions were pooled, and the volume was reduced by using a 100 kDa Amicon Ultra tube (Merck KGaA) and centrifuged at 3000 ***g*** per 3 min.

### Size and concentration determination of parasite‐derived EV by nano‐flow cytometry

Nano‐flow cytometry was performed using a Flow NanoAnalyzer (NanoFCM Co., Ltd, Nottingham, UK) equipped with 488 and 638 nm lasers for characterisation of parasite‐derived EV. Calibration was done with 200 nm polystyrene beads (NanoFCM Co. Ltd.), which had a known concentration of 2.08 × 10^8^ particles·mL^−1^ and were used to reference particle concentration. Monodisperse silica beads (NanoFCM Co., Ltd) of various sizes (68, 91, 113, and 155 nm) served as size standards. To establish the background signal, freshly filtered (0.1 μm) 1× TE buffer at pH 7.4 (Lonza, Basel, Switzerland) was analysed, and these values were subtracted from all subsequent measurements. EV samples were diluted in filtered (0.1 μm) 1× TE buffer, and the particle concentration and size distribution were calculated using NanoFCM software (NF Profession V2.0). Data was collected for 1 min at a sample pressure of 1.0 kPa.

### Protein extraction, SDS/PAGE and immunoblotting

Proteins extracted from *T. gondii*‐derived EV were conducted by following [38]. Cells were sonicated (20 s, 5 times) in RIPA buffer (50 mm Tris–HCl, pH 7.4; 1% NP‐40; 0.5% Na‐deoxycholate; 0.1% SDS; 150 mm NaCl; 2 mm EDTA; 50 mm NaF, all Roth, Carl Roth GmbH Co., Karlsruhe, Germany) supplemented with a protease inhibitor cocktail (1 : 200; Sigma‐Aldrich). Cell homogenates were centrifuged (10 000 ***g***, 10 min, 4 °C) to sediment intact cells, nuclei, and detritus. Respective supernatants were collected and estimated for protein content via the BCA Assay Kit (Thermo Fisher Scientific) following the manufacturer's instructions. For western blot assay, samples were diluted in Laemmli‐β‐mercaptoethanol loading buffer (1610747; Bio‐Rad, Feldkirchen, Germany), and boiled (95 °C) for 5 min. SDS/PAGE gels were loaded with 40 μg proteins/slot and separated in 10–15% polyacrylamide gels via electrophoresis (100 V, 1.5 h; tetra system, Bio‐Rad). Proteins were then transferred to polyvinylidene difluoride membranes (Millipore) (300 mA, 2 h at 4 °C). Samples were blocked in 3% BSA in TBS [50 mm Tris‐Cl, pH 7.6; 150 mm NaCl] containing 0.1% Tween (blocking solution); Sigma‐Aldrich] for 1 h at room temperature (RT) and then incubated with primary antibody (anti‐TSG101, ab125011; Abcam, Cambridge, UK) diluted in blocking solution (overnight, 4 °C). Membranes were washed three times with TBS‐Tween 0.1% buffer, and incubated with secondary antibody (anti‐rabbit IgG, 31466; Pierce, Thermo Fisher Scientific, Waltham, MA, USA) in blocking solution for 30 min, at RT. After three washes in TBS‐Tween (0.1%) buffer, signal detection was accomplished by an enhanced chemiluminescence detection system (ECL plus kit; GE Healthcare, Chicago, IL, USA) and recorded using a ChemoDOC Imager (Bio‐Rad). Protein masses were controlled by a protein ladder (PageRuler Plus Prestained Protein Ladder ~ 10–250 kDa; Thermo Fisher Scientific). Protein band intensities were quantified by the fiji gel analyzer plugin [39].

### Immunofluorescence assays

Immunofluorescence protocol was conducted as previously described in Ref. [40]. Human spermatozoan and *T. gondii* tachyzoites were fixed with paraformaldehyde (4%, 15 min, RT; Roth, Carl Roth GmbH Co.), washed thrice with PBS and incubated in blocking/permeabilisation solution (PBS with 3% BSA, 0.1% Triton X‐100; 1 h, RT). Samples were then incubated overnight, at 4 °C, in a humidified chamber, with anti‐*T. gondii* antibody that was raised specifically against RH strain extracts, and recognises *T. gondii* surface proteins (1/100, PA1‐7256; Thermo Fisher Scientific). On the next day, samples were washed with PBS and incubated in a secondary antibody solution (Alexa Fluor 594 1 : 500, anti‐goat; Thermo Fisher Scientific). Cell nuclei were labeled by a DAPI‐supplemented mounting medium (Fluoromount G; Thermo Fisher Scientific).

### Quantitative PCR

The parasitic load of *T. gondii* in DNA samples extracted from testicular and epididymal tissues from six days post inoculation was quantified using quantitative PCR (qPCR) based on a six‐point standard curve. This curve was generated from 1/10 serial dilutions of a standard DNA from the *T. gondii* ME49 strain (inoculated strain), which corresponded to an initial concentration of 4.5 × 10^6^ tachyzoites·mL^−1^. The qPCR was performed using primers specific for a repetitive element (RE) of *T. gondii*, with each sample analysed in duplicate. Amplification was done using primer pair: TgRE‐Fw 5′‐CACAGAAGGGACAGAAGTCGAA‐3′; TgRE‐Rv 5′‐CAGTCCTGATATCTCTCCTCCAAGA‐3′ following the protocol published by Kasper *et al*. [41] and Souza *et al*. [42]. The reaction was carried out using the SensiFAST™ SYBR No‐ROX Kit (Meridian Bioscience, Cincinnati, OH, USA), following the manufacturer's indications. The cycling conditions were as follows: 95 °C for 3 min, 95 °C for 5 s, and 64 °C for 30 s for 40 cycles, with fluorescence data collected after each cycle. The parasitic load was evaluated using 100 ng of total DNA per sample and normalised to the original DNA concentration of each sample. Samples corresponded to the DNA extracted from the entire organ in all cases.

### Image acquisition and reconstruction

Fluorescence images were acquired using the same protocol described in Ref. [43] with ReScan Confocal microscope instrumentation (RCM 1.1 Visible, Confocal.nl) equipped with a fixed 50 μm pinhole size and combined with an Eclipse Ti2‐A inverted microscope (Nikon, Amstelveen, the Netherlands) which included a motorised Z‐stage (DI1500; Nikon). The RCM unit was connected to a Toptica CLE laser with the following excitations: 405/488/561/640 nm. Images were taken via a sCMOS camera (PCO edge) using a CFI Plan Apochromat 60× lambda‐immersion oil objective (NA 1.4/0.13; Nikon). The setup was operated by the nis‐elements software (version 5.11; Nikon). Images were acquired via z‐stack optical series with a step size of 0.15 microns depth to cover all structures of interest within analysed host cells. Z‐series were displayed as maximum z‐projections. Identical brightness and contrast conditions were applied for each data set within one experiment using fiji software [39].

### Quantification of intracellular ROS production

Intracellular ROS production was quantified in *T. gondii* tachyzoites confronted with pre‐loaded spermatozoa with 10 μm DCFH‐DA (2,7‐dichlorodihydrofluorescein diacetate) for 30 min at 37 °C. After 10 min of confrontation, samples were analysed by a BD Accuri C6 Plus Flow Cytometer (Becton‐Dickinson, Heidelberg, Germany). Cells were gated according to their size and granularity; only morphologically intact sperm cells were included in the analysis. All analyses were performed by the software flowjo v.10 (Ashland, OR, USA). To block ROS production, pre‐treatments of sperm with *N*‐acetyl‐l‐cysteine, NAC (50 μm NAC, 10 min, 37 °C, ab143032; Abcam) were performed before exposure to *T. gondii* tachyzoites.

### DNA fragmentation and apoptosis/necrosis detection in human spermatozoa

DNA fragmentation was assessed by the detection of acridine orange‐derived fluorescence. First, human spermatozoa were incubated with 5 μm acridine orange in sterile PBS for 15 min at 37 °C and washed by centrifugation at 300 ***g*** per 8 min. Stained human spermatozoa were then confronted with *T. gondii* tachyzoites (MOI 1 : 0.5) for 10 min and samples were analysed by a BD Accuri C6 Plus Flow Cytometer (Becton‐Dickinson).

Apoptosis and necrosis were detected by Annexin V/PI labelling (ab14085; Abcam), following the manufacturer's instructions. Spermatozoan were washed by centrifugation, at 300 ***g*** for 8 min, after the staining. They were then confronted with *T. gondii* tachyzoites (MOI 1 : 0.5) for 10 min and analysed by a BD Accuri C6 Plus Flow Cytometer (Becton‐Dickinson).

### Assessment of spermatozoa acrosome reactions

Detection of acrosome reaction was performed by staining human spermatozoa with fluorescein isothiocyanate (FITC)‐labeled *Pisum sativum* agglutinin (PSA; Sigma Chemical Co., St. Louis, MO, USA). The propidium iodide (PI) staining was used to distinguish dead from live sperm. First, spermatozoa were incubated with 1 μg·mL^−1^ PNA and PI for 15 min at 37 °C. Samples were washed out by centrifugation at 300 ***g*** per 8 min. Loaded spermatozoa were confronted with *T. gondii* tachyzoites (MOI 1 : 0.5) for 10 min at 37 °C. Samples were then observed by a BD Accuri C6 Plus Flow Cytometer (Becton‐Dickinson).

### Statistical analysis

All data were expressed as mean ± SD from at least three independent experiments. When the two groups were compared, a Mann–Whitney test was performed (Figs 3A, 4A,B, 5B, and 6). When three or more experimental groups were compared, a Kruskal–Wallis one‐way analysis of variance was applied. Significance was defined as *P* ≤ 0.05. All graphs and statistical analyses were performed using graphpad prism9 software (Merck KGaA).

## Conflict of interest

The authors declare no conflict of interest.

## Author contributions

MEF, RS, and ZDV contributed to conceptualisation. LR‐B, LT‐H, MHNA, and ZDV contributed to main experimentation. UG contributed to SEM and TEM analysis. CP and EPS contributed to EVs analysis. MEF and ZDV contributed to formal analysis. ZDV contributed to statistical analyses. AT, CH and MEF contributed to resources and funding. MEF, RS and ZDV contributed to data curation. ZDV and MEF contributed to writing the original draft. All authors contributed to review and editing.

## Peer review

The peer review history for this article is available at https://www.webofscience.com/api/gateway/wos/peer‐review/10.1111/febs.70097.

## Supporting information


**Fig. S1.** Standard curve for quantitative PCR analysis.
**Fig. S2.** Schematic representation of the flow cytometry gating used in samples depicted in Fig. 4D.

## Data Availability

Supplementary figures and legends informed in the manuscript text are attached to the publication as Supporting Information.
