# Supplementary material for: Adverse impact of acute Toxoplasma gondii infection on human spermatozoa
Source: FEBS J. 2025 May 3;292(17):4720–36. doi: 10.1111/febs.70097 (PMC12414867; doi:10.1111/febs.70097)
Supplement: Supplementary file 1 — Fig. S1. Standard curve for quantitative PCR analysis. Fig. S2. Schematic representation of the flow cytometry gating used in samples depicted in Fig. 4D. [file FEBS-292-4720-s001.pdf]

A

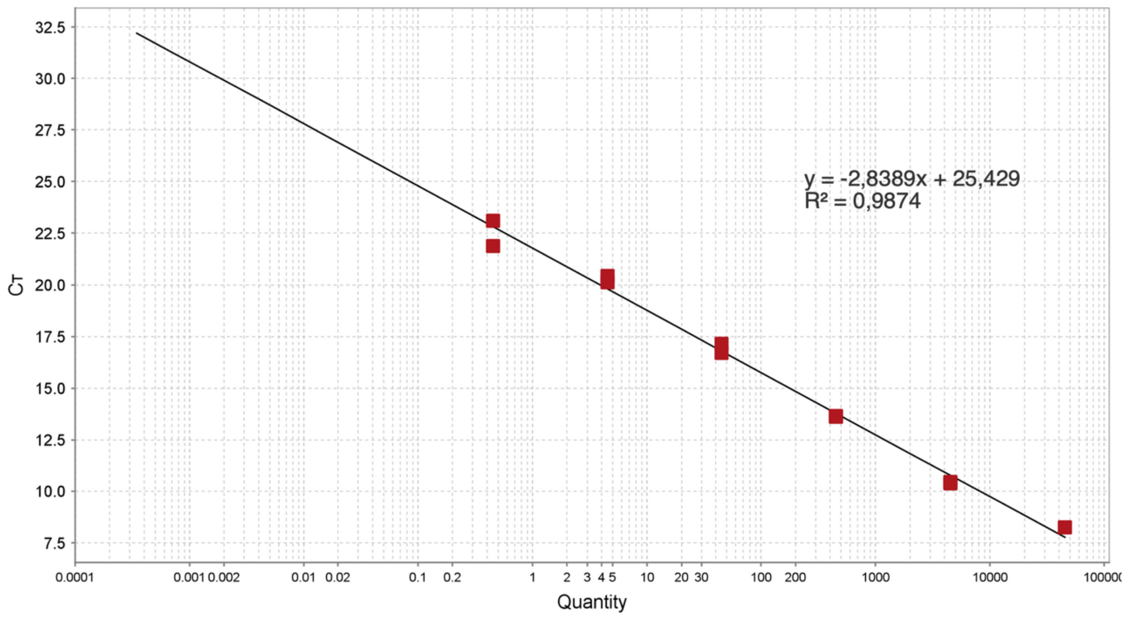

B.

Sample	Average CT (2 replicates)	Estimated parasite load in 100 ng of sample	Sample concentration ng/uL	volume used in qPCR	Estimated total Parasite Load (50 uL)	Average parasite load/ organ
Inf. Left Epid	21,322	1,721848739	157	0,6369427	135,16513	**154,8025**
Inf. Left Test	22,931	1,045798319	213	0,4694836	111,37752	
Inf. Right Epid	20,1	2,235294118	203,6	0,4911591	227,55294	
Inf. Right Test	22,034	1,422689076	204	0,4901961	145,11429	
Uninf. LE	33,92	-3,57142857	348	0,2873563	-621,42857	
Uninf LT	38,45	-5,47478992	93,3	1,0718114	-255,39895	

Supplementary Figure S1. A. Standard curve for quantitative PCR analysis.
A standard curve was generated by plotting the cycle threshold (CT) values against the known number of parasites from which DNA was extracted. The CT values were obtained from serial dilutions of the parasite DNA, and the curve was used to quantify the number of parasites in unknown samples based on their CT values**. B. Estimation of parasite load in the organ based on qPCR analysis.** The table presents the estimated number of parasites in the whole organ, calculated from the CT values obtained through quantitative PCR (qPCR) analysis. The CT values from the samples were referenced against the standard curve shown in A to determine the parasite DNA quantity. The extrapolated values consider organ-specific parameters, and sample dilution, as detailed in the table. These estimates represent the total parasite burden in the organ, providing a quantitative measure of infection.

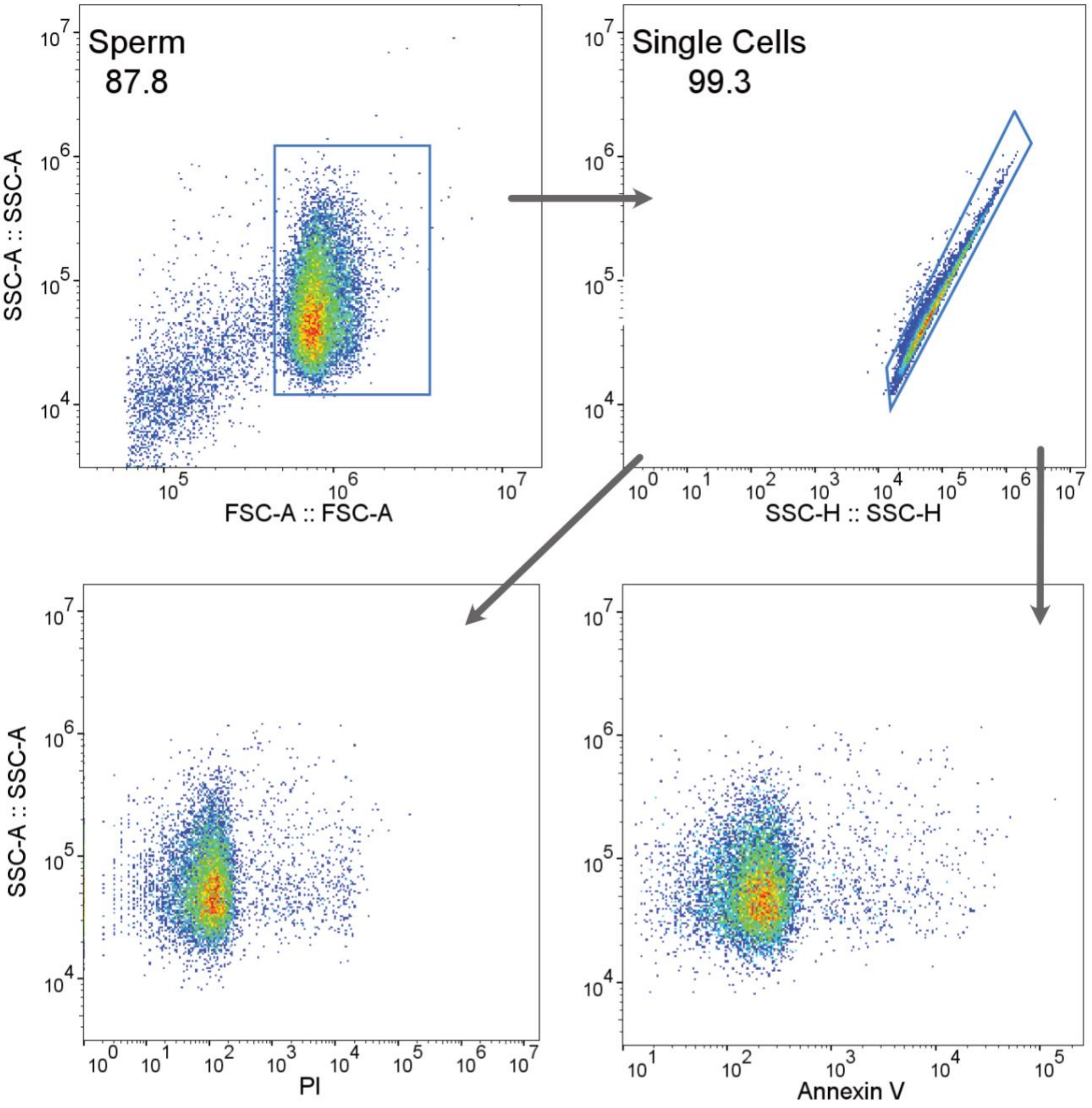

Supplementary Figure S2. Schematic representation of the flow cytometry gating used in samples depicted in Figure 4D. PI and Annexin V stainings were analysed independently to observe necrosis or apoptosis induced by the confrontation between human sperm cells and *T. gondii* tachyzoites.
